# Post-traumatic distal ulnar bifurcation in children: a case report

**DOI:** 10.1186/s12891-023-06494-8

**Published:** 2023-05-30

**Authors:** Jia Wu, Yong Zhu, Zhang-Yuan Lin, Shao-Hai Lin, Shu-Shan Zhao, Liang Cheng, Bu-Hua Sun, Hai-Tao Long

**Affiliations:** Department of Orthopaedics, Xiangya Hospital, Central South University, Changsha, 410008 China

**Keywords:** Galeazzi aequivalent fracture, Conservative treatment, Joint dislocation, Ulna Fractures

## Abstract

**Background:**

Galeazzi fracture dislocation is a compound injury that encompasses fractures of the distal third of the radius and dislocation of the distal radial ulnar joint (DRUJ). Clinically, this condition is rare and often leads to distal ulnar bifurcation. In previous similar reports, patients were effectively managed through surgery.

**Case presentation:**

In this case report, we describe an 11-year-old male child who presented with an ulnar bifida following trauma to the hand, and was treated with manipulation and conservative treatment without surgery. A follow-up performed over the years demonstrated that the patient recovered well, and had normal wrist movements without significant pain, and the patient expressed great satisfaction.

**Conclusions:**

Ulnar diaphyseal fracture may occur in children or adolescents due to injuries, and may be accompanied with manipulation and repositioning. Conservative treatment can be applied to avoid the trauma associated with surgery especially in the absence of severe joint mobility impairment with good outcomes.

## Background

Galeazzi aequivalent fracture dislocation is a compound injury that comprises fractures of the distal third of the radius and dislocation of the distal radial ulnar joint (DRUJ), which is prevalent among adults [1]. In children or adolescents, Galeazzi equivalent fracture dislocation manifests as distal ulnar epiphyseal separation rather than true distal radial ulnar dislocation (DRUJ) [2, 3]. This Galeazzi aequivalent fracture in fact represents a Salter Harris type I or II (or in rare instances a Salter -Harris type IV) injury. Some small epiphyseal injuries may be difficult to detect using imaging techniques and are often overlooked [4]. Here, we report a case of an abnormal distal ulnar bifurcation after trauma with a “Y” shape on imaging. The first case was reported in 2013 by Jones et al. and was successfully treated via surgical operation [5]. In the present report, the patient was treated for bifid ulna conservatively and followed up for 7 years, hence our case presents the entire progression of the patient who received conservative treatment, which is now functioning normally in the wrist.

## Case presentation

**Fig. 1:**
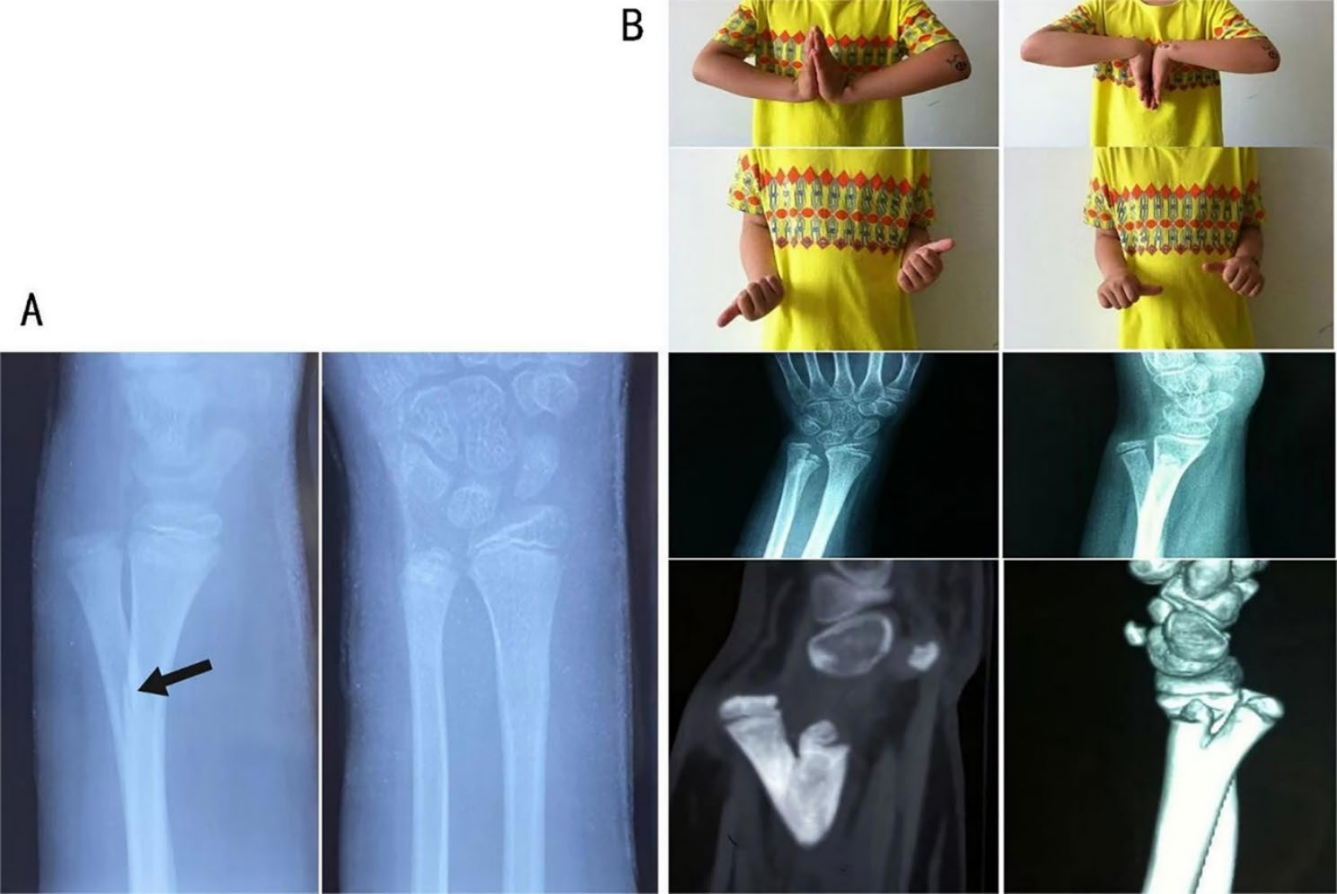
(**A**) X-ray of the patient at the time of injury, greenstick fracture of the left radius (Arrow) with possible dislocation of the distal ulna at the DRUJ. (**B**) The patient’s hand movement, X-ray and CT 3D reconstruction 2 months after the injury

The patient in this case was an 11-year-old male who fell while running and injured his left wrist(We have obtained the consent of the patients and their families regarding sharing information associated with the patient). An X-ray examination at the local hospital revealed a fracture of the distal radius and dislocation of the distal ulnar radial joint. The doctor at the hospital administered the patient 2 manipulations and plaster fixation. Two months later, the patient presented to our hospital due to local swelling and pain. An initial examination of the injury site found no signs of neurovascular damage, but there was pain at the left upper ulnar radial joint, dorsal ulnar bony projection of the left wrist with slight local swelling and significant tenderness. The left wrist joint had nearly unrestricted extension and flexion, but its rotational ability, particularly supination, was limited (Fig. 1).

**Fig. 2:**
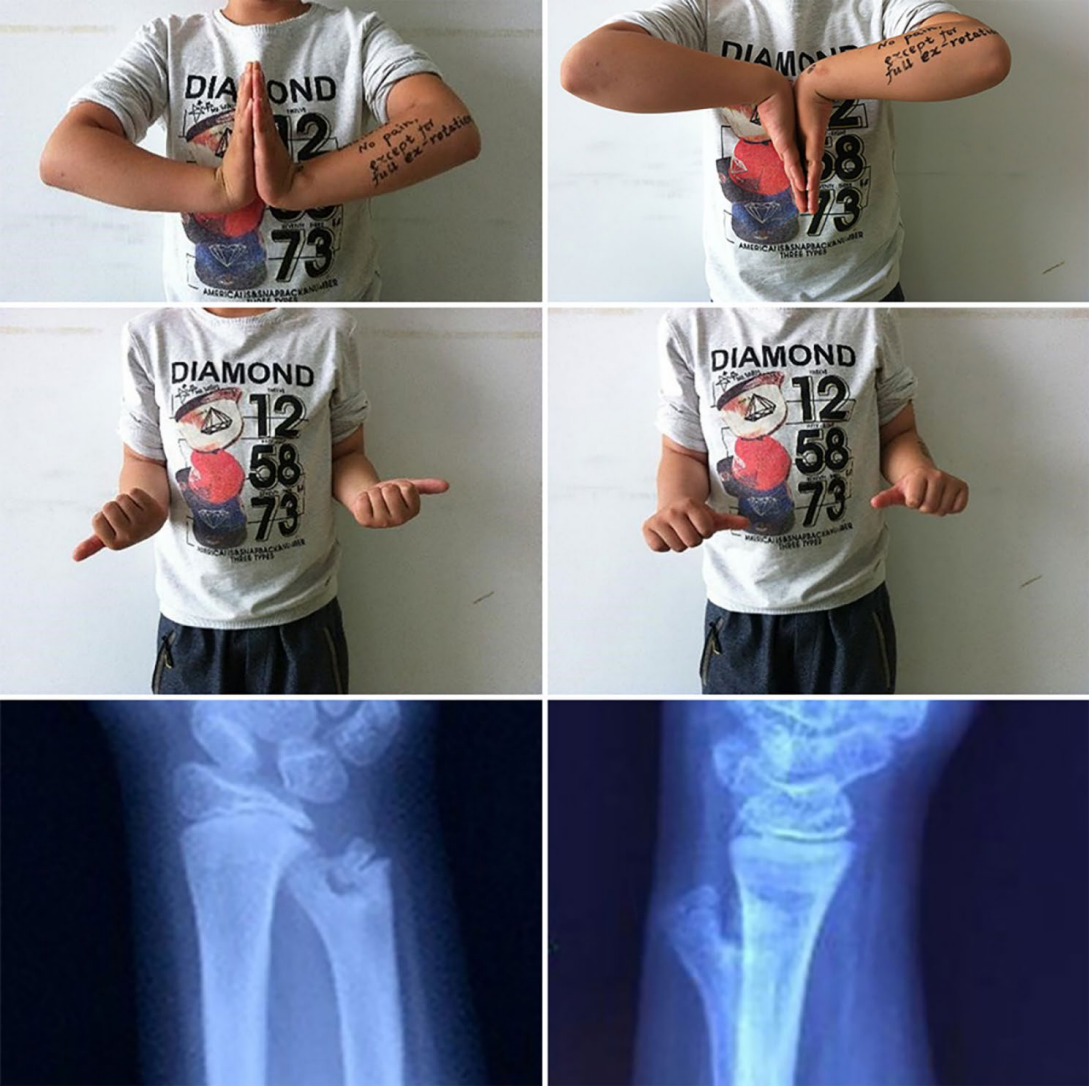
Functional activity and X-ray of the patient’s hand at 7 months post-injury follow-up

We performed an X-ray of the injured site and found that the distal left ulna was split and a new ulna was growing. With the consent of the patient’s family, we performed a CT examination of the area which clearly revealed the deformity was more clearly (Fig. 1). The new ulna grew inwards, was slightly smaller than the original ulna and did not have a complete articular surface, while the normal original ulna protrudes dorsally and laterally with an intact articular surface and a “Y” shaped distal ulna. The patient exhibited normal wrist flexion and extension, but had limited rotation. Following a discussion, the patient’s parents declined surgical treatment. Consequently, we recommended functional exercise and regular follow-up to manage the condition. After a period of seven months, the patient returned to us as a result of experiencing pain. Examination showed that the showed that the pain in the left upper ulnar radius had almost disappeared and the rotation of the left wrist had improved, but there was still pain on extreme supination. The x-ray showed that the distal ulnar bifurcation was still visible, but the two bifurcated ulnae were close to each other and the base was decreased compared with that at 2 months after injury (Fig. 2).

**Fig. 3:**
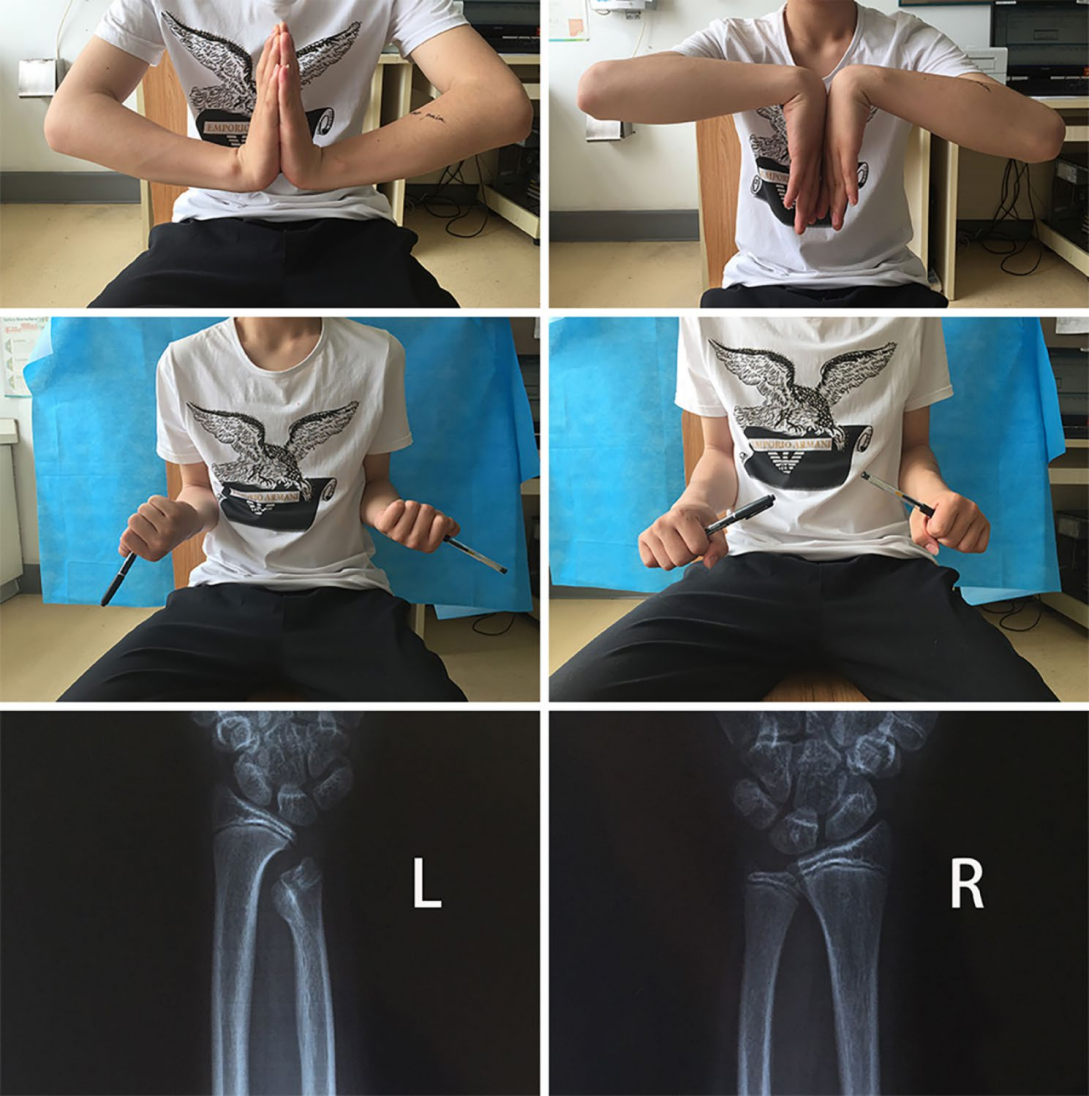
Movement and X-rays at 3 years of follow-up

**Fig. 4:**
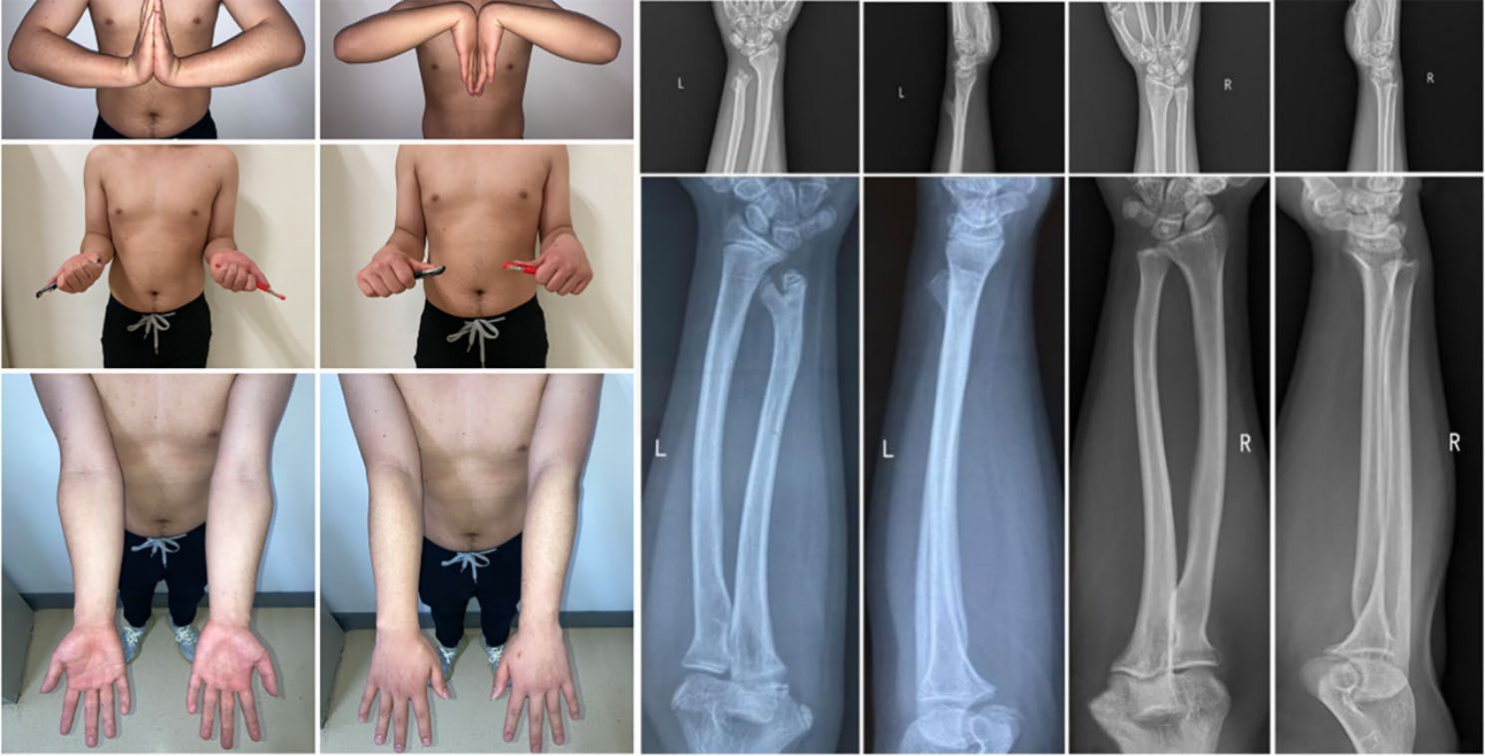
Movement and X-rays at 7 years of follow-up

The patient presented to our hospital at 3 years (Fig. 3) and 7 years (Fig. 4) after the injury. At the last follow-up, the patient’s status was that of a university student, not yet in the workforce. The patient’s left wrist function recovered satisfactorily and did not interfere with his daily life or physical activity, including playing basketball, push-ups and lifting heavy objects. A physical examination revealed that the length of the forearms were equal, the left elbow joint was normal, the left wrist deformity was minimal, the left ulnar styloid process was not prominent, there was no obvious local tenderness, the left wrist extension and flexion and rotation range of activities were normal. However, there was mild pain around the ulnar styloid process on extreme posterior rotation of the left wrist. The X-ray showed that the distal ulna was shortened and bifurcated, but the ulnar bifurcation was atrophied and smaller than previously, the ulnar styloid process was deformed and enlarged, the inferior ulnar radial joint was dislocated, the distal ulna did not participate in the composition of the radial carpal joint, and there was no obvious deformity of the radius.

When performing activities of daily living (ADLs), the normal functional range of wrist motion is 5 degrees of flexion, 30 degrees of extension, 10 degrees of radial deviation and 15 degrees of ulnar deviation [6, 7]. During the follow-up visits, we recorded the patient’s range of motion including wrist flexion and extension, ulnar and radial deviation of the wrist, and anterior/posterior rotation of the forearm (Table 1). The wrist function was rated following the criteria proposed by Krimmer et al. [8].

**Table 1:** Range of motion of the patient’s left wrist at post-injury follow-up

Visit	Extension (°)	Flexion (°)	Ruler deviation (°)	Radial deviation (°)	Supination (°)	Pronation (°)	Krimmer score
2mo	80	85	10	5	30	50	60
7mo	90	87	12	6	70	75	70
3y	95	98	20	8	120	78	90
7y	95	100	22	10	125	80	95

## Discussion

The first such case of Galeazzi aequivalent fracture dislocation was reported by Jones et al. in 2013 [5], in which a 19-year-old man who had undergone surgery for a left wrist injury 12 months later presented with loss of internal and external rotation. Examination revealed a bifurcation of the distal ulna, which they understood at the time to be an occult ulnar injury following an injury that was difficult to detect on imaging. This may have led to an osteochondroma-like lesion growing towards the joint and forming a second ulnar head. In the end, a surgical procedure was performed to remove the head of the distal end of one of the ulna bones. However, the finding of osteochondroma-like lesion has since been questioned, and one such case has been reported and discussed in detail. They argued that the previous claim was questionable due to various reasons. Consequently, they suggested that the new ulna was probably the result of a periosteal cuff injury, followed by subperiosteal ossification and a bifid ulna which was surgically treated [9]. The treatment options for bifid ulna have been explored in recent literature [10]. The authors propose two surgical approaches, i.e. removal of the palmar limb or the dorsal limb. They also suggest that the time of injury is a crucial factor, and that anatomical reconstruction (resection of the palmar limb) should be conducted as early as possible, and a simple dorsal limb resection can be performed if the injury is longer or if a corrective radial osteotomy has been carried out [10]. With regard to the mechanism of formation of the new ulna, we also believe that it was due to subperiosteal ossification. At the time of injury, the patient fell and developed the injury and wrist rotation, causing the ulnar periosteum to tear away owing to the pulling of the interosseous membrane of the forearm. These alterations were not fully aligned at the time of the manipulation, which lead to the tearing of the periosteum and subperiosteal ossification to form a new ulna. In previous cases, surgical treatment was applied [5, 9, 10]. In the present report, the patient was 11 years old at the time of the injury and had no significant restriction of hand movement after the injury and showed only mild restriction of rotational function after the new ulna had grown out. Subsequent follow-up showed that the conservative treatment had effectively restored functional movement to normal state.

## Conclusion

We report a case of post-traumatic distal ulnar bifurcation, which may also be referred to as a bifurcated ulna, and demonstrate for the first time the course and prognosis of the bifurcated ulna after conservative treatment. In children and adolescents presenting with a bifid ulna after a Galeazzi equivalent fracture, conservative treatment after repositioning may be attempted in patients whose motor function is not significantly limited to avoid the trauma associated with surgery. we think prospective multi-center studies are required to find out more about the most efficient treatment of Galeazzi aequivalent injury in adolescents.

## Data Availability

All data generated or analyzed during this study are included in this published article.

## Abbreviations

DRUJ: Distal radial ulnar joint
CT: Computed tomography
ADLs: Activities of daily living
mo: Months
y: Years
